# Novel mutations in the *RS1* gene in Japanese patients with X-linked congenital retinoschisis

**DOI:** 10.1038/s41439-018-0034-6

**Published:** 2019-01-08

**Authors:** Hiroyuki Kondo, Kazuma Oku, Satoshi Katagiri, Takaaki Hayashi, Tadashi Nakano, Akiko Iwata, Kazuki Kuniyoshi, Shunji Kusaka, Atsushi Hiyoshi, Eiichi Uchio, Mineo Kondo, Noriko Oishi, Shuhei Kameya, Atsushi Mizota, Nobuhisa Naoi, Shinji Ueno, Hiroko Terasaki, Takeshi Morimoto, Masayoshi Iwaki, Kazutoshi Yoshitake, Daisuke Iejima, Kaoru Fujinami, Kazushige Tsunoda, Kei Shinoda, Takeshi Iwata

**Affiliations:** 1 Department of Ophthalmology, University of Occupational and Environmental Health, Kitakyushu, Japan; 2 Department of Ophthalmology, The Jikei University School of Medicine, Tokyo, Japan; 3 Department of Ophthalmology, Kindai University Faculty of Medicine, Osakasayama, Japan; 4 Department of Ophthalmology, Fukuoka University, Fukuoka, Japan; 5 Department of Ophthalmology, Mie University Graduate School of Medicine, Tsu, Japan; 6 Department of Ophthalmology, Nippon Medical School Chiba Hokusoh Hospital, Chiba, Japan; 7 Department of Ophthalmology, Teikyo University, Tokyo, Japan; 8 Department of Ophthalmology, Miyazaki University, Miyazaki, Japan; 9 Department of Ophthalmology, Nogoya University Graduate School of Medicine, Nagoya, Japan; 10 Department of Applied Visual Science, Osaka University Graduate School of Medicine, Osaka, Japan; 11 Department of Ophthalmology, Aichi Medical University, Nagakute, Japan; 12 Division of Molecular and Cellular Biology, National Institute of Sensory Organs, National Tokyo Medical Center, Tokyo, Japan; 13 Division of Vision Research, National Institute of Sensory Organs, National Tokyo Medical Center, Tokyo, Japan; 14 Department of Ophthalmology, Saitama Medical University, Moroyama, Japan

**Keywords:** Mutation, Medical genetics

## Abstract

X-linked congenital retinoschisis (XLRS) is an inherited retinal disorder characterized by reduced central vision and schisis of the macula and peripheral retina. XLRS is caused by mutations in the *RS1* gene. We have identified 37 different mutations in the *RS1* gene, including 12 novel mutations, in 67 Japanese patients from 56 XLRS families. We present clinical features of these patients in relation to the associated mutations.

X-linked congenital retinoschisis (XLRS) is an inherited retinal disorder that affects central vision and manifests in early childhood^1^. XLRS is the most common inherited retinal disorder; its highest reported prevalence is 14 per 10,000 individuals in Finland^1^. XLRS is characterized by foveal retinoschisis, which occurs in nearly 100% of patients, whereas peripheral schisis is present in 50% of XLRS patients^1^. Neuronal dysfunction, manifested as a reduction in the b-wave/a-wave ratio of dark-adapted electroretinograms (DA-ERGs), is also a characteristic of XLRS, although the detection rate for this sign has varied^2,3^. Clinical diagnosis is not easily determined in certain cases because of the wide range of phenotypes, which may include macular and retinal degeneration and secondary complications such as vitreous hemorrhage and retinal detachment; thus, genetic diagnosis is helpful.

XLRS is caused by mutations in the *RS1* gene^4^, which encodes retinoschisin, a 24-kDa retina-specific protein secreted by photoreceptors and bipolar cells. Retinoschisin functions as a cell adhesion protein that maintains the synaptic structure of the retina^5^. To date, according to the Human Gene Mutation Database (HGMD; 2018.2 version, https://portal.biobase-international.com), 251 different mutations in this gene are known to cause XLRS. Because of variations in phenotypes among and within families, the genotype–phenotype relationship has not yet been definitively established ^6,7^.

We conducted a multicenter observational study at 12 institutions located throughout Japan; this study was approved by the ethics committee of each institution. Signed written informed consent was obtained from all participants or their parents.

Sixty-seven Japanese patients from 56 families with XLRS were studied (Table 1). All patients were male, and their average age was 19.1 years (range: 2 months to 57 years). XLRS was diagnosed based on retinal findings, including the presence of foveal schisis with or without peripheral schisis and a reduced b-wave/a-wave ratio on dark-adapted ERGs as well as family history^1^. The 56 patients included 14 patients with familial XLRS and 42 patients with sporadic XLRS. Medical records were reviewed for all patients who had been identified as carriers of mutations in the *RS1* gene.

**Table 1:** Mutations in the *RS1* gene and clinical features in patients with congenital retinoschisis

Family no	Patient ID	Kinship	Age	Famial/ sporadic	Mutation				Ocular features							
					Exon/intron	Nucleotide change (NM_000330.3)	Amino acid change	Novel/ reported (ID)	Visual acuity	Refraction of spherical equivalent (D)	Retinoschisis		Electroretinogram			Comment
											Foveal	Peripheral	ERG a-amplitude (μV)	b/a ratio	Light intensity (cds/m^2^)	
1	KS0001	Proband	4mo	Sporadic	1	c.35T>C	p.Leu12Pro	Novel	0.02/NA	−0.375/+0.375	−/ +	+/+	46/38.3	0.61/0.66	30	B) Vx
2	F111	Proband	2	Sporadic	1	c.38T>C	p.Leu13Pro	rs104894935	NA/NA	NA/NA	+ / +	+/+	NA/NA	NA/NA		R) VH
3	J0968	Proband	36	Sporadic	1	c.49G>T	p.Glu17*	Novel	0.8/0.3	+ 0.625/−0.375	+ / +	−/−	312.5/226	0.78/0.93	3	
4	RS30-1	Proband	8	Sporadic	2–3	exon2-3 del		Novel	0.3/0.3	+ 4/ + 3.625	+ / +	+ / +	227/167	0.91/0.95	30	L) Vx
5	J0913	Proband	6	Sporadic	IVS2	c.78+2T>C	USD	Novel	0.5/0.7	+ 1.125/ + 0.75	+ / +	+ / +	267.5/NA	0.56/NA	200	
6	RS13-1	Proband	4	Familial	3	exon3 del		Undetermined	0.4/0.2	+ 5.5/ + 2	+/ +	+ / +	490/416	1.11/0.97	^b^	L) VH, Vx, B) retinal fold
6	RS13-2	Sibling	11	Familial	3	exon3 del		Undetermined	1.2/1.2	+ 0.75/ + 0.75	+/ +	−/−	NA/NA	NA/NA		
7	RS14-1	Proband	3	Sporadic	3	c.98G>A	p.Trp33*	CM141023^a^	0.6/0.06	+ 5.25/ + 6.5	+ / +	+ /+	345/274	0.96/1.2	^b^	
8	RS01-1	Proband	16	Familial	3	c.175T>G	p.Cys59Gly	Novel	0.5/0.02	0/−7.5	+ / +	+ /+	467/360	0.55/0.7	3	L) VH
8	RS01-2	Sibling	23	Familial	3	c.175T>G	p.Cys59Gly	Novel	0.5/0.5	+ 0.5/ + 0.5	−/−	−/−	314/331	0.3/0.25	3	
8	RS01-3	Sibling	20	Familial	3	c.175T>G	p.Cys59Gly	Novel	0.4/0.4	−0.25/0	+ / +	+ /+	375/374	0.58/0.47	3	
9	RS11-1	Proband	12	Familial	3	c.175T>G	p.Cys59Gly	Novel	0.05/0.8	+ 2/−0.25	+ / +	+ /+	383/403	0.74/0.83	3	R) Retinal hole
9	RS11-2	Sibling	7	Familial	3	c.175T>G	p.Cys59Gly	Novel	0.4/0.3	+ 0.5/−0.25	+ / +	−/ +	457/401	0.9/0.76	3	
10	J0381	Proband	17	Sporadic	3	c.185_186insT	p.Glu62Aspfs*24	Novel	0.3/0.3	−3/−2.5	+/ +	−/−	129/146	1.11/1.16	3	
11	J0673	Proband	13	Sporadic	4	c.214G>A	p.Glu72Lys	rs104894928	0.8/0.7	−2/−2	+ / +	+ /+	185/195	0.79/0.76	3	
12	J1033	Proband	13	Sporadic	4	c.214G>A	p.Glu72Lys	rs104894928	0.5/0.9	−1/−0.5	+ / +	−/−	229/252	1.31/1.28	3	
13	J1062	Proband	32	Sporadic	4	c.214G>A	p.Glu72Lys	rs104894928	0.5/0.4	−2.375/ + 0.875	+ / +	+ /+	119.6/134.3	0.76/0.86	3	
14	RS04-1	Proband	16	Familial	4	c.214G>A	p.Glu72Lys	rs104894928	0.3/0.15	−1.75/−2.5	+ / +	+ /+	362/344	0.55/0.67	200	
14	RS04-1	Sibling	9	Familial	4	c.214G>A	p.Glu72Lys	rs104894928	0.7/0.06	−1.25/NA	+ / +	+ /+	NA/NA	NA/NA		
15	KINKI-113	Proband	50	Sporadic	4	c.214G>A	p.Glu72Lys	rs104894928	0.2/0.2	−2.75/−1.625	N/N	N/N	370/370	0.78/0.8	30	
16	NMSCHH011-01	Sibling	42	Familial	4	c.214G>A	p.Glu72Lys	rs104894928	CF/0.2	NA/ + 0.25	N/ +	N/+	NA/344.5	NA/0.76	10	
16	NMSCHH011-02	Proband	47	Familial	4	c.214G>A	p.Glu72Lys	rs104894928	0.15/0.3	+ 0.25/ + 0.75	−/−	+ /+	308/243	1.07/1.18	10	
17	RS12-1	Proband	3	Sporadic	4	c.218C>T	p.Ser73Leu	Novel	NA/NA	NA/NA	+ / +	−/+	~0/~0	NA/NA		L) retinal degeneration
18	J0256	Proband	18	Sporadic	4	c.266A>G	p.Tyr89Cys	rs61752060	0.4/0.08	+ 0.75/ + 1.5(IOL)	+ / +	+ /+	250/168	0.72/0.61	3	L) Vx, IOL
19	J1224	Proband	6	Sporadic	4	c.266A>G	p.Tyr89Cys	rs61752060	0.08/0.5	+ 6/ + 6	+ / +	+ /-	NA/NA	NA/NA		
20	Teik1051	Proband	49	Sporadic	4	c.266A>G	p.Tyr89Cys	rs61752060	0.1/0.1	+ 5/ + 6	−/−	+ /+	309.5/137	0.59/0.53	10	L) retinal degeneration
21	Teik1103	Proband	10	Sporadic	4	c.266A>G	p.Tyr89Cys	rs61752060	0.03/0.6	+ 2.75/ + 0.25	+ / +	−/−	38.3/35.1	1.25/1.12	3	
22	KINKI-107-1	Proband	26	Familial	4	c.267T>A	p.Tyr89*	rs61752061	0.4/0.3	+ 3/ + 2.75	+ / +	+ /+	420/400	0.71/0.7	30	
22	KINKI-107-2	Sibling	25	Familial	4	c.267T>A	p.Tyr89*	rs61752061	0.3/0.3	−0.5/−1	+ / +	−/−	300/280	1.27/1.29	30	
23	RS21-1	Proband	11	Sporadic	4	c.285delG	p.Trp96Glyfs*30	Novel	0.2/0.6	−1.5/0.75	+ / +	+ /+	503/na	0.75/NA	200	R) Vx
24	RS16-1	Proband	6	Sporadic	4	c.301G>C	p.Ala101Pro	rs61752066	0.5/0.7	+ 0.75/ + 1	+ / +	−/−	NA/345	NA/0.63	10	B) ODRL
25	RS17-1	Proband	4	Sporadic	4	c.304C>T	p.Arg102Trp	rs61752067	0.03/0.15	NA/ + 3.125	+ / +	+ /+	301/289	0.83/1.06	^b^	
26	RS31-1	Proband	3	Familial	4	c.304C>T	p.Arg102Trp	rs61752067	0.4/NA	+ 1.375/NA	+ / +	+ /+	346/349	0.96/0.96	200	L) Vx
26	RS31-2	Sibling	2mo	Familial	4	c.304C>T	p.Arg102Trp	rs61752067	NA/NA	NA/NA	+ / +	−/−	NA/NA	NA/NA		
27	Teik1153	Proband	45	Sporadic	4	c.304C>T	p.Arg102Trp	rs61752067	0.032/0.5	+ 6/ + 2	1/ +	−/−	146.5/304	0.69/0.62	^b^	
28	J1330	Proband	5	Sporadic	4	c.305G>A	p.Arg102Gln	rs61752068	0.4/0.4	+ 9.5/ + 8	+ / +	+ /+	462/607.8	0.79/0.74	200	
29	RS23-1	Proband	1	Familial	4	c.305G>A	p.Arg102Gln	rs61752068	0.7/0.06	+ 6.625(IOL)/ + 5(IOL)	+ / +	+ /+	43.8/43.8	0.55/0.69	200	L) Vx, IOL
29	RS23-2	Sibling	4	Familial	4	c.305G>A	p.Arg102Gln	rs61752068	0.3/0.12	NA/NA	+ / +	+ /+	NA/NA	NA/NA		
30	RS08-1	Proband	2	Sporadic	4	c.326G>C	p.Gly109Ala	Novel	0.2/0.3	+ 2.25/ + 2.75	+ / +	+ /+	184/208	0.87/0.77	^b^	R) VH, Vx
31	MIYA003-1	proband	16	Familial	4	c.330T>A	p.Cys110*	rs1801161	0.7/1.2	−0.5/0	+ /+	−/−	377.8/302.3	0.78/0.72	3	B) retinal fold, Vx, glaucoma
31	MIYA003-2	Sibling	14	Familial	4	c.330T>A	p.Cys110*	rs1801161	0.9/0.7	−0.25/ + 0.75	+ /+	−/−	87.8/83	0.77/0.74	3	
32	RS18-1	Proband	8	Familial	5	c.404G>A	p.Gly135Glu	Novel	0.4/0.3	+ 4.475/ + 4.625	+ / +	−/−	NA/NA	NA/NA		
33	J0690	Proband	25	Familial	5	c.417G>T	p.Gln139His	Novel	0.3/0.4	−0.75/ + 0.25	−/−	−/−	NA/NA	NA/NA		B) ODRL
34	J0852	Proband	7	Sporadic	5	c.422G>A	p.Arg141His	rs61752159	0.7/0.4	+ 1.5/ + 1.875	+ / +	−/−	NA/NA	NA/NA		
35	MIE52	Proband	53	Familial	5	c.438G>C	p.Glu146Asp	rs61753163	0.5/0.4	−0.5/0	−/−	−/−	270/294	0.81/0.71	30	B) macular degeration
36	J0892	Proband	19	Sporadic	IVS5	c.522+1G>A	USD	rs281865348	0.4/0.3	+ 4.25/ + 4.625	+ / +	+ /+	172.1/130.6	0.45/0.5	3	
37	NHO1025	Proband	54	Sporadic	IVS5	c.523-1G>A	USD	Novel	0.2/0.15	−3.5/−4.125	+ / +	−/−	343.25/391.25	0.9/0.93	10	
38	RS25-1	Proband	8	Sporadic	6	c.544C>T	p.Arg182Cys	rs61753171	0.1/1.2	+ 2.625/−0.125	+ / +	+ /+	283/318	0.74/0.85	200	R) Vx
39	RS26-1	Proband	3mo	Sporadic	6	c.544C>T	p.Arg182Cys	rs61753171	0.5/0.2	+ 1(IOL)/−1(IOL)	+ / +	+ /+	175.5/200.8	0.69/0.64	30	R) Vx IOL
40	RS27-1	Proband	9mo	Sporadic	6	c.544C>T	p.Arg182Cys	rs61753171	0.3/0.3	−0.25/−2.75(aphakia)	+ / +	+ /+	NA/NA	NA/NA		L) Vx
41	J1461	Proband	38	Sporadic	6	c.544C>T	p.Arg182Cys	rs61753171	0.4/0.4	−6.5/−7.125	+ / +	+ /+	360.5/348.3	0.78/0.68	200	
42	KIN	Proband	33	Sporadic	6	c.574C>T	p.Pro192Ser	rs61753174	0.8/0.9	−0.225/−0.25	+ / +	−/−	290/280	0.86/0.82	30	
43	RS07-1	Proband	16	Sporadic	6	c.589C>T	p.Arg197Cys	rs281865354	0.1/0.09	+ 10/ + 9	−/−	+ /+	292/237	1/0.47	200	
44	MIE49	Proband	43	Sporadic	6	c.589C>T	p.Arg197Cys	rs281865354	0.2/0.3	+ 2/ + 1.5	+ / +	−/−	290/280	0.45/0.5	30	
45	RS32-1	Proband	5mo	Sporadic	6	c.589C>T	p.Arg197Cys	rs281865354	Follow/Follow	NA/NA	+ / +	+ /+	257/313	0.95/0.74	200	B) VH, R) Vx
46	RS10-1	Proband	28	Sporadic	6	c.590G>A	p.Arg197His	rs281865355	0.06/0.08	NA/NA	+ / +	+/+	NA/NA	NA/NA		
47	RS19-1	Proband	43	Familial	6	c.598C>T	p.Arg200Cys	rs281865357	0.2/0.05	+ 2.5/ + 2.5	+ / +	+ /+	281/355	1.35/0.69	^b^	
48	RS15-1	Proband	6	Sporadic	6	c.599G>A	p.Arg200His	rs281865358	0.7/0.7	+ 0.5/−0.575	+ / +	−/−	273.5/248	0.83/0.72	^b^	
49	NTMC218	Prpband	43	Sporadic	6	c.599G>A	p.Arg200His	rs281865358	0.1/0.1	+ 0.25/ + 0.25	+ / +	+ /+	268/175.3	0.29/0.86	10	B) Retinal hole
50	RS29-1	Proband	1	Sporadic	6	c.608C>T	p.Pro203Leu	rs104894930	0.4/NLP	NA/NA(aphakia)	+ / +	+ /+	NA/NA	NA/NA		L) proliferative vitreoretinopathy
51	J0903	Proband	52	Sporadic	6	c.608C>T	p.Pro203Leu	rs104894930	0.5/0.3	−1.125/−1.25	−/−	−/−	174.3/292	0.83/0.77	3	B) macular degeration
52	J0371	Proband	57	Sporadic	6	c.625C>T	p.Arg209Cys	rs281865361	CF/0.01	NA/0.875	+ / +	−/−	114/218	1.25/0.67	3	
53	J0640	Proband	31	Sporadic	6	c.625C>A	p.Arg209Ser	Novel	0.3/1.2	−1/−0.5	+ /−	−/−	295/279	0.93/0.99	^b^	
54	RS06-1	Proband	49	Sporadic	6	c.638G>A	p.Arg213Gln	rs281865364	0.15/0.04	−0.5/0	−/−	+ /+	312/332	0.77/0.59	^b^	
55	RS05-1	Proband	7	Sporadic	6	c.657C>G	p.Cys219Trp	CM101549^a^	0.3/0.3	+ 0.25/−0.5	−/−	+ /+	344/400	0.69/0.71	^b^	
56	MIYA020-1	proband	9	Familial	6	c.667T>C	p.Cys223Arg	rs104894929	0.4/0.5	−1/−1.5	+ / +	+/+	436/399.5	0.53/0.52	3	L) retinal fold, Vx
56	MIYA020-2	Sibling	12	Familial	6	c.667T>C	p.Cys223Arg	rs104894929	0.4/0.4	−1.5/−1	+ /+	−/+	342/382.8	0.55/0.52	3	L) retinal fold, Vx

*B* both eyes, *CF* counting finger, *L* left eye, *IOL* intraocular lens, *mo* month-old, *NA* not available, *ODRL* Oguchi disease-like retinal reflex, *R* right eye, *USD* undetermined splicing defect, *VH* vitreous hemorrahge, *Vx* vitrectomy, + present, − absent

^a^SNP (rs) ID is unavailable and ID of the Human Gene Mutation Database is shown

^b^20J (data are not interchangable with unit of cds/m^2^)

Genomic DNA was extracted from peripheral blood using DNA extraction kits or manual extraction with ethanol. Polymerase chain reaction (PCR) followed by Sanger sequencing was performed on 56 samples for six coding exons of the *RS1* gene unless whole-exon deletions were detected via PCR. In brief, oligonucleotide primers for the flanking intron/untranslated region sequences were designed, and PCR was performed, followed by uni- or bidirectional sequencing depending on the quality of the PCR products. The primer sequences and annealing temperature for PCR for each exon are available on request. The other 11 samples were screened by whole-exome sequencing with at least 30× coverage for all exons. To identify sequence variations, reference sequences of *RS1* (NM_000330.3) were used; variations were numbered based on the cDNA sequence, with +1 corresponding to the first nucleotide of the initiation codon (ATG).

Thirty-seven different mutations in the *RS1* gene were identified in the 56 families, including 26 missense, 4 nonsense, 3 splicing, 1 deletion, 1 insertion, and 2 whole-exon deletion mutations (Table 1). Eleven point mutations were novel mutations, and 24 point mutations had previously been reported, based on the HGMD and one recent report (Table 1)^8^. A whole-exon deletion of exon 3 had been reported^9^, whereas a deletion of exons 2 and 3 has not been reported. In our study, DNA break points were not determined, and it is unknown whether the exon 3 deletion that we observed was identical to the known exon 3 deletion at the DNA level.

The frequency of the 11 novel point mutations was assessed using public domain databases. None of these variants were found in human genome variation databases for the Japanese population (the Human Genetic Variation Database (HGVD), http://www.hgvd.genome.med.kyoto-u.ac.jp/) or other population databases, such as the 1000 Genomes Project database (http://www.internationalgenome.org/1000-genomes-browers), the Exome Aggregation Consortium (ExAC) database (http://www.exac.broadinstitute.org), and the 6500-exome database of the NHLBI-ESP project (ESP6500, http://evs.gs.washington.edu/EVS/). The pathogenicity of the seven novel missense mutations was predicted in silico by nine programs and via folding energy assessments^6,10–17^. Overall, all variants were considered to be pathogenic (Table 2).

**Table 2:** Pathogenicity assessment of the novel missense mutations in the *RS1* gene

Nucleotide change	Amino acid change	Folding energy value^6^ (assessment)	Polyphen2 HumDIV^10^ (cutoff = 0.85)	GERP++^11^ (cutoff = 2)	REVEL^12^ (cutoff = 0.5)^a^	M-CAP^13^ (cutoff = 0.025)	CADD^14^ phred (cutoff = 15)^b^	PROVEAN^15^ (cutoff = −2.5)	SIFT^15^ (cutoff = 0.05)	Mutation Accessor^16^ (cutoff = 1.9)	FATHMM^17^ (cutoff = −1.5)
c.35T>C	p.Leu12Pro	NA	0.984	5.69	0.701	0.782	25.100	−1.120	0.003	2.095	−5.160
c.175T>G	p.Cys59Gly	0.04 (weak)	0.999	5.15	0.868	0.844	23.900	−2.850	0.000	2.610	−5.010
c.218C>T	p.Ser73Leu	0.22 (weak)	0.953	5.43	0.825	0.896	27.200	−4.460	0.002	3.925	−5.630
c.326G>C	p.Gly109Ala	1 (severe)	1.000	5.43	0.740	0.921	28.000	−0.860	0.233	0.780	−4.810
c.404G>A	p.Gly135Glu	1 (severe)	0.953	4.91	0.985	0.943	27.100	−5.540	0.001	3.515	−5.610
c.417G>T	p.Gln139His	1 (severe)	0.996	2.14	0.920	0.949	23.800	−4.830	0.001	4.435	−5.600
c.625C>A	p.Arg209Ser	0.74 (moderate)	1.000	5.63	0.770	0.909	25.700	−0.200	0.044	1.695	−5.220

Underlined values are indicated as “pathogenic” according to the cutoff values (refs. ^10–17^)

*NA* not applicable

^a^75.4% of disease mutations but 10.9% of neutral variants

^b^≤1% percentile highest scores

Seven of the known mutations were detected in more than one family; in particular, p.Glu72Lys, p.Tyr89Cys, p.Arg182Cys, p.Arg102Trp, p.Arg197Cys, p.Arg200His, and p.Pro203Leu were observed in 6, 4, 4, 3, 3, 2, and 2 families, respectively. These mutations have previously been reported in the same population and in other populations^4,7,9^. Mutation hot spots were suggested instead of founder effects as an explanation of these mutations.

Overall, the clinical findings of this study were consistent with those of earlier reports, although detailed phenotype–genotype relationships remain undetermined^1,3,6,7^.

Of the 109 phakic eyes for which refractive error (in spherical equivalents) was measured, there were 60 (55.0%), 5 (4.6%), and 44 (40.4%) hypermetropic, emmetropic, and myopic eyes, respectively (Table 1). For the hypermetropic eyes, the refractive error ranged from 0.25 to 10.0 diopters (D), and the average error was + 2.7 D. For the myopic eyes, the refractive error ranged from −0.125 to −7.5 D, and the average error was −1.6 D. The average difference in refractive error between the two eyes was 1.0 D for 51 patients.

For 125 eyes, the decimal best-corrected visual acuity varied from counting fingers to 1.2, with a median of 0.3. For 131 eyes for which retinal status was determined, retinoschisis was present in the macula in 110 eyes (84.0%) and in the periphery in 88 eyes (61.8%).

DA-ERGs were recorded in 104 eyes using different stimulus intensities; intensities that tended to be higher than those recommended in the standard protocol from the International Society of Clinical Electrophysiology of Vision were used for certain patients^18^. Negative ERGs were more frequently observed in this study (84.6%, Table 1) than in earlier studies^3^, likely due to the use of higher-intensity light stimuli^19^.

The observed retinal complications included a need for pars plana vitrectomy (*N* = 16); macular or retinal degeneration, including Oguchi disease-like retinal surface abnormalities (*N* = 6); vitreous hemorrhage (*N* = 5); retinal folds (*N* = 4); and congenital glaucoma (*N* = 1).

We sought to establish a possible phenotype-genotype relationship for eyes with truncation mutations (i.e., nonsense, splicing, deletion, insertion, or exon deletion mutations) as opposed to missense mutations. The newly identified mutations do not appear to produce distinct clinical phenotypes compared with reported mutations. However, patients with novel missense mutations did present at an earlier age than those with reported missense mutations (data not shown).

Foveal schisis was more frequently found in eyes with truncation mutations than in those with missense mutations (100% versus 78%, *P* = 0.0035, Supplemental Table 1). It is possible that nearly normal foveal structure can only be seen in eyes with missense mutations^20^. Peripheral schisis was found in 50% and 67% of eyes with truncation and missense mutations, respectively (*P* = 0.107).

Compared with eyes with missense mutations, eyes with truncation mutations showed larger b-waves (*P* = 0.023) and higher b/a ratios (*P* = 0.019) on DA-ERG, whereas no significant difference was observed for the mean a-wave amplitude (Supplemental Table 2). Differences in patient age, visual acuity, refractive error, and light stimulus settings for DA-ERGs were not significant.

Vincent et al.^20^ reported that truncation mutations were associated with poor visual acuity and a higher probability of a b/a ratio < 1.0. Our data yielded contradictory results, with higher b-wave amplitude and a greater b/a ratio in eyes with truncation mutations than in eyes with missense mutations. One possible reason for this discrepancy is that the patients with truncation mutations presented at a younger age, which tends to be associated with better preservation of ERG findings^21^. Nonetheless, our study implies that it will be difficult to determine a phenotype–genotype relationship using ERGs.

This study has limitations. Because of the retrospective nature of this investigation, in which only mutation-proven cases were selected, the identification rate of the *RS1* gene in XLRS has not been determined. A history of clinical findings, including vitreous hemorrhages, may have been missed in certain cases due to only reviewing medical records.

In summary, this study was the largest survey of patients with mutations in the *RS1* gene in the Japanese population. The progress of gene therapy for XLRS has reached the clinical trial stage, and exact genetic determinations for each patient could lead to more efficient future treatments^22^.

## Supplementary information

Supplement tables 1 and 2

## Data Availability

The relevant data from this Data Report are hosted at the Human Genome Variation Database at 10.6084/m9.figshare.hgv.2408 10.6084/m9.figshare.hgv.2411 10.6084/m9.figshare.hgv.2414 10.6084/m9.figshare.hgv.2417 10.6084/m9.figshare.hgv.2420 10.6084/m9.figshare.hgv.2423 10.6084/m9.figshare.hgv.2426 10.6084/m9.figshare.hgv.2429 10.6084/m9.figshare.hgv.2432 10.6084/m9.figshare.hgv.2435 10.6084/m9.figshare.hgv.2438 10.6084/m9.figshare.hgv.2441 10.6084/m9.figshare.hgv.2444 10.6084/m9.figshare.hgv.2447 10.6084/m9.figshare.hgv.2450 10.6084/m9.figshare.hgv.2453 10.6084/m9.figshare.hgv.2456 10.6084/m9.figshare.hgv.2459 10.6084/m9.figshare.hgv.2462 10.6084/m9.figshare.hgv.2465 10.6084/m9.figshare.hgv.2468 10.6084/m9.figshare.hgv.2471 10.6084/m9.figshare.hgv.2474 10.6084/m9.figshare.hgv.2477 10.6084/m9.figshare.hgv.2480 10.6084/m9.figshare.hgv.2483 10.6084/m9.figshare.hgv.2486 10.6084/m9.figshare.hgv.2489 10.6084/m9.figshare.hgv.2492 10.6084/m9.figshare.hgv.2495 10.6084/m9.figshare.hgv.2498 10.6084/m9.figshare.hgv.2501 10.6084/m9.figshare.hgv.2504 10.6084/m9.figshare.hgv.2507 10.6084/m9.figshare.hgv.2510 10.6084/m9.figshare.hgv.2513
