# Supplementary material for: Novel mutations in the RS1 gene in Japanese patients with X-linked congenital retinoschisis
Source: Hum Genome Var. 2019 Jan 8;6:3. doi: 10.1038/s41439-018-0034-6 (PMC6325138; doi:10.1038/s41439-018-0034-6)
Supplement: Supplementary file 1 — Supplement tables 1 and 2 [file 41439_2018_34_MOESM1_ESM.docx]

| **Supplemental table 1. Comparison of frequency of foveal and peripheral schisis in eyes with truncation mutations and missense mutations in the *RS1* gene** | | | |
| --- | --- | --- | --- |
|  | Truncation mutation | Missense mutation | p |
| Presence of foveal schisis | 28 (100%) | 71 (78%) | 0.0035* |
| Presence of peripheral schisis | 14 (50%) | 61 (67%) | 0.120** |
| *Fisher exact test, **Chi-square test | |  |  |

| **Supplemental table 2. Comparison of dark-adapted electroretinograms in eyes with truncation mutations and missense mutations in the *RS1* gene** | | | |
| --- | --- | --- | --- |
|  | Truncation mutation | Missense mutation | p* |
| n | 24 | 71 |  |
| Age | 19.2±14.3 | 26.2±17.7 | 0.088 |
| LogMAR | 0.46±0.28 | 0.68±0.56 | 0.067 |
| Refaction | 1.11±3.02 | 0.24±2.89 | 0.216 |
| Dark-adapted ERG |  |  |  |
| a wave amplitude (uV) | 283±123 | 271±106 | 0.645 |
| b wave amplitude (uV) | 252±126 | 198± 88 | 0.023 |
| b/a ratio | 0.88±0.23 | 0.75±0.23 | 0.019 |
| light intensity setting |  |  |  |
| 3/10/30/200/# (cds/㎡) | 10/2/6/2/4 | 28/7/14/10/12 | 0.937 |
| *Chi-square test was used for light intensity of ERG and Student t-test for the others. | | | |
| #20J (data are not interchangable with unit of cds/㎡) | | |  |
| ERG: electroretinogram, LogMAR: logarithm of minimum angle resolution | | |  |
